# Partial Pulpotomy with Mineral Trioxide Aggregate in Permanent Incisors with Complicated Crown Fracture: 5-Year Follow-Up

**DOI:** 10.1155/2020/8855331

**Published:** 2020-09-05

**Authors:** Isabel Gomes

**Affiliations:** Department of Oral Rehabilitation, Faculty of Dental Medicine, University of Lisbon, Lisbon, Portugal

## Abstract

Traumatic dental injuries are highly prevalent among children. This article describes a case report of a patient who has experienced dental trauma at 8 years old, which has led to enamel-dentine fracture with pulp exposure in the left central incisor and crown-root fracture with pulp exposure in the right central incisor. Partial pulpotomy was performed with the aim of maintaining the neurovascular bundle, thus allowing normal radicular formation. During follow-up 5 years later, teeth were asymptomatic and with no evidence of radiolucent lesions in radiographic examinations. This report demonstrates that traumatic fractures with pulp exposure can be treated effectively by the described technique.

## 1. Introduction

Traumatic dental damages happen with high incidence in youngsters and teenagers, corresponding to 5% of all damages that individuals are searching treatment for [1]. A 12-year literature review acknowledges that 25% of all school children experience dental damage [2]. In permanent dentition, crown fracture with pulp exposure corresponds to 6.4-18.3% of the injuries [3–5].

In immature permanent teeth with traumatic exposed pulps, partial and total pulpotomy are the treatment of choice [6–15]. Pulpotomy is the amputation of part of the dental pulp, permitting the rest the tooth to continue to live and retain its habitual function. In partial pulpotomy (Cvek pulpotomy), 2 mm of the pulp below the exposure are removed, leaving a wound of healthy pulp, where dressing should be placed over. Unlike total pulpotomy, Cvek pulpotomy implies the conservation of cell-rich coronal pulp tissue which is more prone to favor recovery than the radicular pulp [7]. Mineral trioxide aggregate (MTA), among many materials, has been used as a substitute agent to the gold standard calcium hydroxide- (CH-) based cement in pulpotomy treatments [8, 9, 16]. Partial pulpotomy with CH has reported success rates between 13 and 96% [6, 17]. However, recent research has identified that MTA has produced the creation of more regular and denser dentin bridges than CH [16, 18, 19]. Less porosity (tunnel defects) in dentin bridges provides more effective sealing against microorganism penetration and this is probably the reason for a more successful outcome [8, 9, 16, 20].

This clinical report is aimed at describing a 5-year follow-up of two traumatized immature permanent maxillary incisor teeth with pulp exposure.

## 2. Case Report

**Figure 1:**
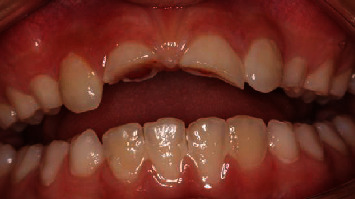
Fracture with pulp exposure in both incisors.

**Figure 2:**
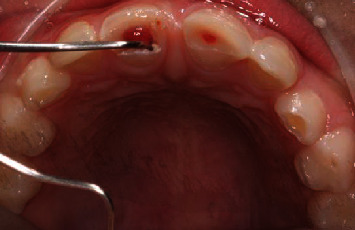
Midcrown fracture with small pulp exposure in tooth no. 21 and crown-root fracture with protrusive pulp reaction in tooth no. 11.

**Figure 3:**
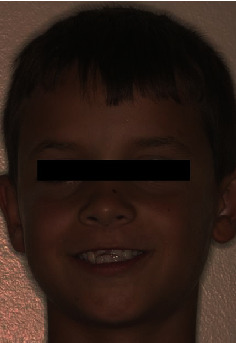
Frontal view of the patient.

**Figure 4:**
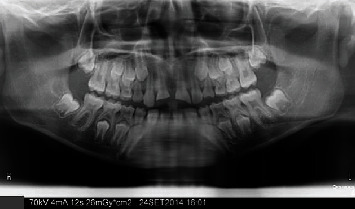
Panoramic radiography 3 days after trauma.

An 8-year-old male patient was sent for management after 3 days of an enamel-dentine-pulp fracture in the upper left central incisor (number 21) and crown-root fracture with pulp exposure in the upper right central incisor (number 11) (Figures 1–4). Trauma occurred during a football match. The patient denied spontaneous pain on presentation. Through intraoral examination, pulp exposure was diagnosed in both incisors, as well as mild pain to percussion, absence of pain to palpation, absence of periodontal pockets higher than 3 mm, mobility class I, and positive response to cold test in both incisors that were performed on the labial surface. The size of the pulp lesion was registered (nearly 2 mm in tooth 21 and 4 mm in tooth 11) with proliferated pulp reaction in tooth 11. Radiographic examination showed no root fractures or periradicular radiolucency.

**Figure 5:**
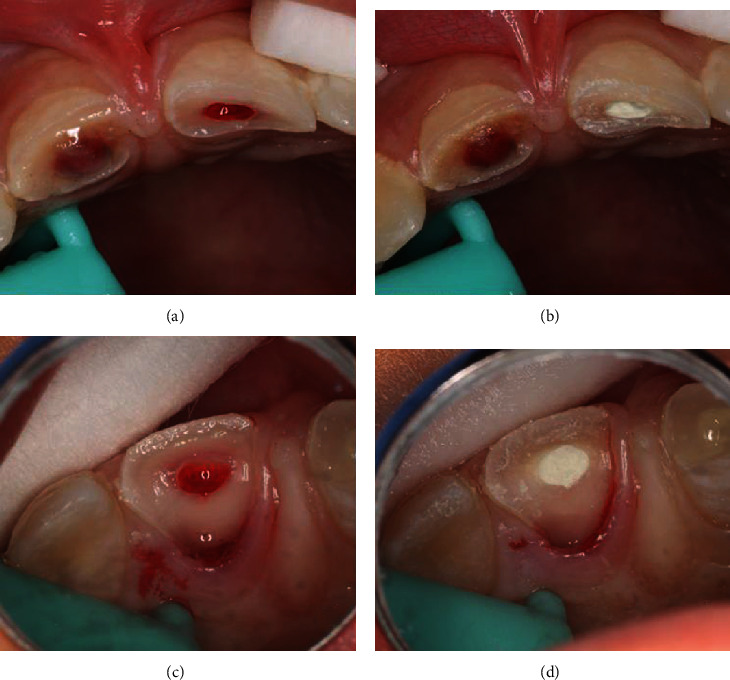
Pulpotomy of tooth no. 21 (a and b) and tooth no. 11 after removal of the palatine tooth fragment and after partial pulpotomy (c) with MTA application (d).

**Figure 6:**
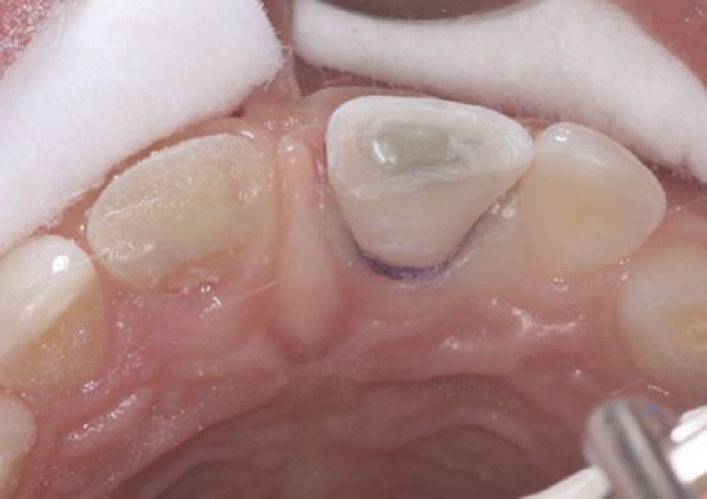
2 weeks later, the glass ionomer was partially removed and direct composite restoration was concluded.

The pulpotomy procedure was explained to the parents and the patient who agreed to comply with it. The concerned area was anesthetized with local infiltration of 1.7 ml lidocaine HCL 2% with 1 : 100000 epinephrine. After local anesthesia, the palatine fragment was removed from tooth 11. After this, nearly 2 mm depth of visible pulp tissue and adjacent dentin of both incisors (11 and 21) were removed, with a sterile diamond cylindrical drill on a high-speed handpiece with water irrigation. A hole about 2 mm deep remained, enough to hold the wound dressing and sealing material. Bleeding was controlled with water squirted out of a syringe, and MTA (Pro Root, Dentsply, Tulsa Dental, OK, USA) was applied with a spatula-shaped hand instrument, followed by wet cotton pellets used to adjust it onto the visible pulp space. A layer of MTA 2 mm thick was positioned over the exposed pulp tissue. After 10 minutes, a glass ionomer (Vitrabond Plus, 3M ESPE, USA) was applied over the MTA, and the patient was dismissed (Figure 5). After 2 weeks, the patient was asymptomatic, with no pain to palpation, absence of periodontal pockets higher than 3 mm, mobility class I, and positive response to the cold test. The glass-ionomer restoration was partially removed, and directly bonded composite restoration was performed (IPS Empress Direct, IvoclarVivadent, Schaan, Liechtenstein) with gum retraction (Ultrapak 1, Ultradent, Utah, USA) (Figure 6).

**Figure 7:**
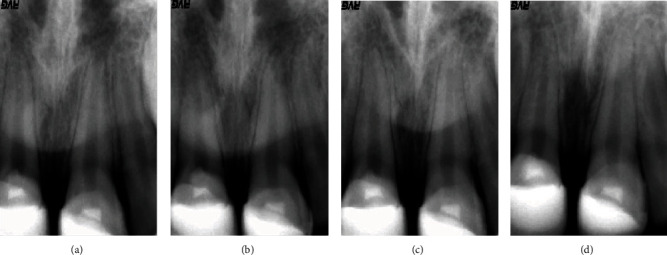
Periapical radiography, at 2 months (a), 1 year (b), 2 years (c), and 3 years (d) after trauma.

**Figure 8:**
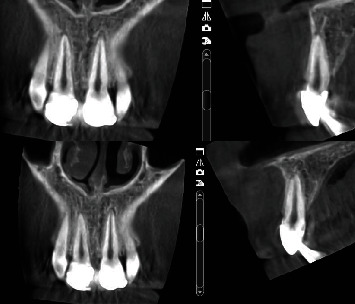
CBCT 5 years after trauma with a normal appearance.

Teeth were followed clinically and radiographically two weeks after treatment and subsequently during the first year at three months, and afterwards, at six-month intervals. During the 6-month follow-up and from 1 to 5 years, the situation remained unchanged with no evidence of radiolucent lesions in radiographic examinations (Figure 7). Cone-beam computerized tomography (CBCT) was used to monitor healing during the 5-year follow-up, allowing 3D visualization of the periapical area and discarding the existence of root resorption (Figure 8).

**Figure 9:**
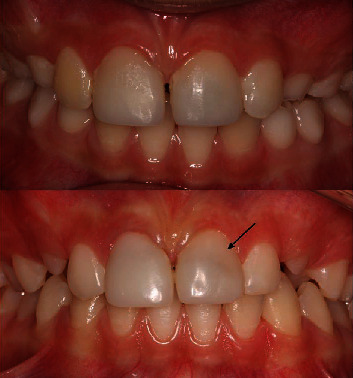
Follow-up after 6 months (up) and 3 years (down) with cervical discoloration in tooth no. 21.

Follow-up shows a continuation of root development and the creation of hard tissue at the site of the incision (Figure 7). Light discoloration in the crown of 21 was evident at 3 years follow-up (Figure 9), but it did not disturb the patient or his family, so it was not addressed.

## 3. Discussion

Guidelines should support dentists in decision making and in providing the best care to patients. Accordingly, the International Association of Dental Traumatology (IADT) and the American Academy of Pediatric Dentistry (AAPD) have issued a consensus statement after analyzing dental publications and have recommended partial pulpotomy for this clinical situation [12, 13]. This recommendation is based on the fact that every effort must be made to preserve pulpal vitality in the young permanent tooth to guarantee continuous root development and increased resistance of the tooth. Recovery capability after traumatic pulp exposure is well known [12], but what about bacterial penetration in the three-day elapsed time between the accident and treatment? Is partial pulpotomy also recommended when an exuberant proliferated pulp reaction exists? To obtain these answers, we have to find support in clinical evidence. Researchers have reported that neither time interval between injury and treatment nor size (less than 4 mm) of pulpal exposure affects the prognosis of partial pulpotomy with calcium hydroxide dressing [6, 21]. In a clinical record of partial pulpotomy in 60 children's teeth with treatment intervals between 1 h and 90 days, Cvek has established in 1978 that time was not key for the recovery of an initially healthy pulp, grounded on the treatment success percentage of 96.7%. Nevertheless, most of the evaluated teeth were assisted ≤100 h. Other reports suggest that up to a 9-day interval between damage onset and treatment may have minimum consequences in the outcome of Cvek's pulpotomies [7, 14]. Clearly, it is not crucial to execute the treatment of problematic crown fractures promptly after trauma [15, 19], but there are still no research findings that have evaluated the success rate of Cvek pulpotomy in teeth with pulp exposures bigger than 4 mm, and prognosis has not yet been clear.

In this clinical case, we have a young patient with immature roots and open apices. With regard to this, it seems that teeth with open apices have a superior prognosis [14, 15, 22]. The age of the patient may adversely affect the outcome of conservative pulp treatments, since in older patients the pulp is more fibrotic and has reduced ability to recover [23]. Not consistent with these conclusions, de Blanco described a 100% Cvek pulpotomy success rate with CH power executed in 30 teeth, 20 with closed apices and 10 with open apices at the treatment instance, advocating that the outcome of Cvek pulpotomies is not affected either by the existence of an open or closed apex during the treatment.

The level of pulp amputation was close to 2 mm under the level of the exposure, leaving a cavity that could hold the MTA and in accordance with the assumption that inflammatory modifications found in recently injured pulps are likely to be superficial, based on previous studies [21]. Cvek et al. evaluated complicated fractures in primate teeth and discovered that after 3 h, hemorrhage and injury at the odontoblastic level did not surpass 2 mm on the pulp exposure side. After 48 h, it extended from 1.5 to 2 mm, and after 7 days, it extended from 0.8 to 2.2 mm. In pulps exposed for 48 h to 7 days, nearly 36% showed proliferated pulp reactions [21].

Regarding dressing material, MTA has become an alternative, after evidence has shown that it was the most successful pulp-capping substance [8, 9, 20, 24]. In a large randomized clinical trial, higher performance with MTA as a direct pulp-capping dressing, when compared to CH, was confirmed in a follow-up for up to 2 years [8]. On the other hand, Qudeimat et al. in 2017 have found no statistical difference between the success ratio of MTA-treated teeth (93%) and those treated with calcium hydroxide MTA (91%), after partial pulpotomy, in permanent teeth with decay exposures [25]. With regard to MTA disadvantages, discoloring effects are among the most cited [24, 26, 27] and this was acknowledged in this clinical case. Fortunately, reports have detected that most discoloration was due to inner MTA and not to infiltrated dentin [28]. Moreover, it seems that blood constituent penetration in porosities inside MTA may be the main reason for the discoloration and not the type of MTA (gray or white) [29]. Other possible explanations for tooth discoloration may be ferric oxide, bismuth oxide, and magnesium oxide ingredients in the MTA powder. Based on this, and if discoloration arises, when a continuous hard tissue wall is perceived in the radiographs, pigmented MTA can be removed and aesthetics will be improved.

Analyzing similar clinical cases reports, Subay et al. [26] have followed up six immature teeth with MTA pulpotomies after traumatic and mechanical pulp exposures and have found two unsuccessful cases and severe discoloration in all six cases. Borkar and Ataide [30] have followed up four cases of fully matured traumatized maxillary permanent central incisors, which have been treated via biodentine pulpotomy, several days after traumatic pulp exposure. The pulp showed signs of vitality and absence of periapical radiolucency after 18 months in all cases. Neither of these two reports has included a proliferated pulp reaction. In the present case, two immature central incisors have been dealt with partial pulpotomy with MTA and have remained healthy after 5 years. During follow-up, no clinical symptoms were observed, and in radiographs and CBCT, it was possible to notice root development, with a visible dentin bridge formation and no intraradicular or periradicular pathological changes. This case report might be a technically challenging case, especially in young children, but the benefits have outweighed the hurdles and have confirmed the possibility that it can be an option, even in cases of proliferated pulp reaction.

## 4. Conclusion

1. Traumatic pulp exposures can be treated effectively by the abovementioned technique. The use of MTA in traumatized teeth treated with partial pulpotomy has been confirmed.
2. In this case, the presence of hyperplastic pulp did not prevent partial pulpotomy with MTA from having a successful outcome.
3. This method has the benefit of maintaining the neurovascular bundle, allowing normal radicular formation.
